# Azithromycin for uncomplicated severe acute malnutrition: study protocol for a pilot randomized controlled trial

**DOI:** 10.1186/s40814-021-00836-w

**Published:** 2021-04-20

**Authors:** Kieran S. O’Brien, Ali Sié, Clarisse Dah, Millogo Ourohire, Ahmed M. Arzika, Valentin Boudo, Elodie Lebas, William W. Godwin, Benjamin F. Arnold, Catherine E. Oldenburg

**Affiliations:** 1 Francis I Proctor Foundation, University of California, San Francisco, 490 Illinois St, San Francisco, CA USA; 2 Centre de Recherche en Santé de Nouna, Nouna, Burkina Faso; 3 Centre de Recherche et Interventions en Santé Publique, Niamey, Niger; 4 Department of Ophthalmology, University of California, San Francisco, USA; 5 Department of Epidemiology & Biostatistics, University of California, San Francisco, USA

**Keywords:** Severe acute malnutrition, Azithromycin, Amoxicillin, Weight gain

## Abstract

**Background:**

Given the high risk of infectious mortality among children with severe acute malnutrition (SAM), the World Health Organization recommends routine administration of a broad-spectrum antibiotic like amoxicillin as part of the management of uncomplicated SAM. However, evidence for the efficacy of amoxicillin to improve nutritional recovery or reduce mortality has been mixed. With a long half-life and evidence of efficacy to reduce mortality in high-risk populations, azithromycin is a potential alternative to amoxicillin in the management of SAM. In this pilot study, we aim to compare the efficacy of azithromycin to amoxicillin to improve nutritional outcomes in children with uncomplicated SAM.

**Methods:**

This pilot randomized controlled trial will enroll 300 children with uncomplicated SAM from 6 Centre de Santé et de Promotion Sociale in the Boromo health district in Burkina Faso. Eligible children are randomized to receive a single directly observed dose of oral azithromycin or a 7-day course of oral amoxicillin in addition to the standard package of care for uncomplicated SAM. Enrolled children are followed weekly until nutritional recovery, and all children return for a final study visit at 8 weeks after enrollment. Anthropometric indicators, vital status, and clinical outcomes are monitored at each visit and compared by arm. Primary feasibility outcomes include enrollment potential, refusals, loss to follow-up, and completeness of data collection. The primary clinical outcome is weight gain (g/kg/day) over the 8-week study period.

**Discussion:**

This pilot trial will establish the feasibility of conducting a full-scale randomized controlled trial to evaluate alternative antibiotics in this setting and provide preliminary evidence for the efficacy of azithromycin compared to amoxicillin to improve outcomes for children with SAM.

**Trial registration:**

This trial was first registered on clinicaltrials.gov on 26 June 2018 (NCT03568643).

## Background

Children with severe acute malnutrition (SAM) often bear a large burden of infection and related mortality [1]. Malnutrition can suppress the immune system, and children with SAM can present with asymptomatic infections. As a result, the World Health Organization (WHO) recommends routine treatment of children with uncomplicated SAM with a broad-spectrum antibiotic [2]. Randomized controlled trial evidence for this recommendation is mixed. Two studies of amoxicillin as adjunctive therapy for SAM found varying results. In Malawi, routine amoxicillin led to an increased probability of nutritional recovery and decreased probability of mortality [3]. In Niger, there was no effect of routine amoxicillin on either nutritional recovery or mortality [4]. A third study of daily cotrimoxazole for children recovering from complicated SAM found no benefit for nutritional recovery or mortality [5]. These conflicting results may indicate that empiric antibiotic therapy for uncomplicated SAM is unnecessary in some settings or that a different antibiotic class is needed.

The *Macrolides Oraux pour Réduire les Décès avec un Oeil sur la Résistance* trial (MORDOR) demonstrated that biannual mass azithromycin distribution reduced all-cause mortality when distributed at the population level to preschool children, regardless of the presence of clinical signs of infection or other morbidities [6, 7]. While the precise mechanism behind this effect is unclear, it is likely due to an overall reduction in infectious burden and reduced infectious mortality [8]. For example, distribution of azithromycin to preschool children led to reduced malaria parasitemia and reduced mortality from meningitis, dysentery, malaria, and pneumonia [8, 9]. Although MORDOR was not powered to detect an effect in subgroups, the effect was most pronounced in those with higher mortality, including in younger children, in Niger, and among underweight children [6, 10, 11]. This led to the hypothesis that targeting azithromycin to high-risk subgroups may be an effective strategy to prevent mortality. Although at the population level targeting treatment may not reduce as many deaths as mass treatment, individual-level mortality may be reduced with targeted treatment and it may be a more feasible health systems delivery approach [12].

Children with SAM are at increased risk of mortality compared to their well-nourished peers [13]. As a high-risk subgroup, azithromycin may be a particularly beneficial intervention. As an alternative to amoxicillin, azithromycin is attractive because it can be delivered as a single, oral dose due to its long half-life. Here, we describe the design of a pilot randomized trial to compare the effect of azithromycin to amoxicillin on nutritional outcomes in the management of uncomplicated SAM in Burkina Faso. As this type of study has not been conducted in this setting, this pilot trial was designed to establish the feasibility of conducting a larger randomized controlled trial evaluating antibiotics for SAM in this study area in Burkina Faso by determining enrollment potential, refining potential study procedures, and providing data to update assumptions used in sample size calculations.

## Methods/design

### Aims and hypotheses

The overall objectives of this pilot trial are to (1) lay the groundwork for a full-scale randomized controlled trial evaluating different antibiotics for uncomplicated SAM, which includes establishing the feasibility of enrollment and study procedures as well as refining assumptions for sample size calculations, and (2) compare the efficacy of azithromycin to amoxicillin on weight gain and nutritional recovery in the management of uncomplicated SAM. We hypothesize that children randomized to receive azithromycin will have both increased weight gain and increased nutritional recovery compared to amoxicillin 8 weeks after admission to the nutritional program.

### Study design

In this pilot individual-randomized trial, children 6-59 months of age with uncomplicated SAM presenting to nutritional programs at eligible health centers in Boromo District, Burkina Faso will be randomized to a single dose of oral azithromycin or a 7-day course of amoxicillin upon admission to the program. Apart from the administration of antibiotics, all children will receive standard outpatient treatment for uncomplicated SAM as specified in the guidelines of the government of Burkina Faso, which includes therapeutic feeding with ready-to-use therapeutic food (RUTF) [14]. Enrolled children will be followed weekly at each routine follow-up visit until nutritional recovery. All children will return for a final study visit at 8 weeks following enrollment. Anthropometric and vital status data will be collected at each follow-up visit, and weight gain and nutritional recovery over the 8-week period will be compared by arm.

### Study oversight

Ethical approval for this study will be obtained from the Comité d’Ethique du Burkina Faso and the University of California, San Francisco, before study activities begin. Written informed consent will be obtained from a parent or guardian for the participation of each child. At the time of consent, the parent or guardian will be informed that participation is voluntary and that they can withdraw their child at any time and receive the same standard of care outside of the study. An infectious disease specialist at UCSF will serve as the Medical Monitor to provide clinical oversight on the study design and to monitor serious adverse events.

A Data and Safety Monitoring Committee (DSMC) will be empaneled before the study begins to provide independent oversight of data quality and patient safety. The DSMC will include experts in biostatistics, epidemiology, child health, and nutrition. The DSMC will approve the study protocol before the study begins, and the DSMC will monitor study progress, data collection, and adverse events through annual meetings with the study team and review of quarterly progress reports. The DSMC will make recommendations regarding the study protocol and potential modifications as well as the continuation or termination of the study based on safety data. Protocol modifications will be shared with both IRBs and the DSMC.

### Setting

The trial will be conducted at 6 Centre de Santé et de Promotion Sociale (CSPS) that offer nutritional programs for children presenting with SAM from a catchment area of 54 communities in Boromo District, Burkina Faso. CSPSs are government-run integrated health centers linked to and overseen by district hospitals that are primarily nurse-led and provide primary treatment and preventative care. Each CSPS has one severe acute malnutrition day per week, during which children are screened and treated for severe acute malnutrition.

### Recruitment

Each CSPS participating in the study will screen all children presenting to the CSPS for eligibility for the trial. Community-based screening for SAM with referral to the CSPS also occurs through community health workers and the CSPS weekly malnutrition days.

### Eligibility criteria

**Fig. 1:**
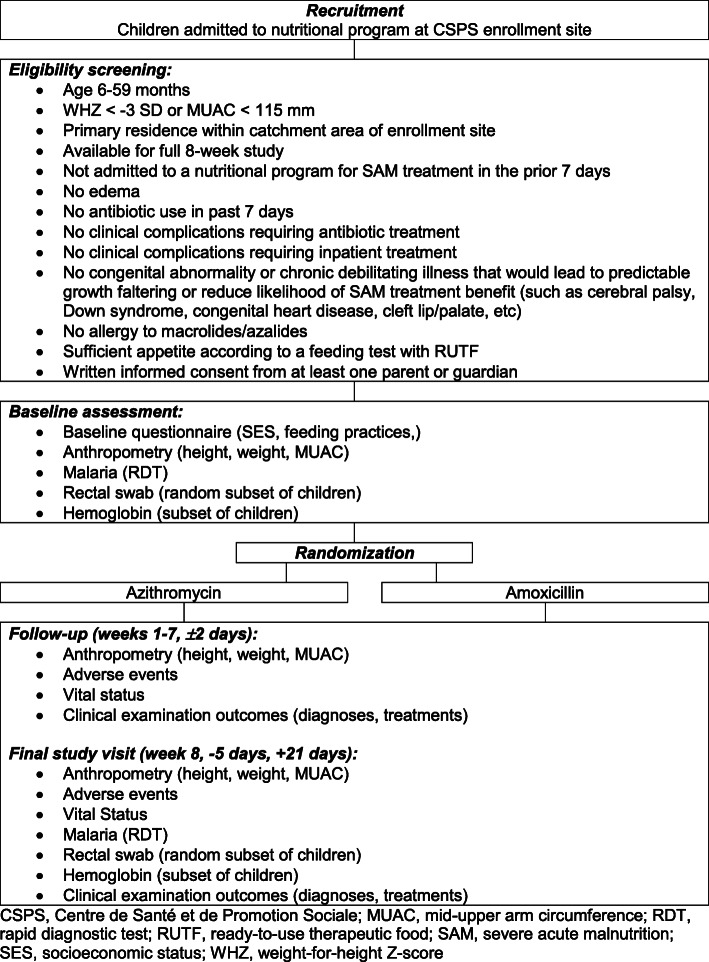
Participant flow diagram with eligibility criteria. CSPS, Centre de Santé et de Promotion Sociale; MUAC, mid-upper arm circumference; RDT, rapid diagnostic test; RUTF, ready-to-use therapeutic food; SAM, severe acute malnutrition; SES, socioeconomic status; WHZ, weight-for-height *Z*-score

For inclusion in the pilot trial, enrollment sites must have seen more than 200 cases of SAM in 2017, be willing to participate in the trial, and have their participation approved by the district. Eligible individuals are age 6–59 months with SAM presenting to an enrollment site during the study period who meet all of the criteria outlined in Fig. 1.

### Randomization and masking

Eligible children will be enrolled by CSPS staff trained for the study. At enrollment, children will be assigned a study identification number using the next unassigned number on a list assigned to the site. After completion of a baseline assessment, children will be randomized to receive either a single dose of directly observed oral azithromycin or a 7-day course of oral amoxicillin.

The randomization sequence was generated by the UCSF investigators using R (R Foundation for Statistical Programming, Vienna, Austria). Children will be randomized in a 1:1 fashion to a single dose of azithromycin or a 7-day course of oral amoxicillin without stratification or blocking. The randomization sequence will be linked to the study identification numbers and implemented in the mobile application used for data collection (CommCare by Dimagi, Cambridge, MA, USA). After enrollment and completion of the baseline assessment, the application will indicate the child’s assigned intervention.

To keep the allocation concealed, only the CSPS nurse administering treatment will have access to the data collection form indicating the randomization allocations. Given the nature of the intervention, participants and study personnel administering treatment will not be masked to treatment assignment. Outcome assessors will be masked to treatment assignment; this will be accomplished by assigning one CSPS nurse to administer treatment and a separate masked study team member to perform outcome assessments, including anthropometry, malaria rapid diagnostic test, and vital status updates.

### Interventions

Children will be randomized to receive a single directly observed dose of oral azithromycin or a 7-day course of oral amoxicillin. Azithromycin is administered as a single weight-based dose at the time of enrollment in oral suspension form for children. Dosing follows the WHO recommendations for treatment of active trachoma, which indicates a single dose of 20 mg/kg in children (up to the maximum adult dose of 1g). Individuals who are allergic to macrolides/azalides will not be treated. Azithromycin is purchased locally by CSPS study team members (Azithrin oral suspension 200 mg/5ml, Strides Shasun Ltd, Bangalore, India). Oral amoxicillin (80 mg/kg of body weight per day, divided into two daily doses) is administered for 7 days. The first dose will be directly observed and administered by the CSPS nurse, who will teach the caregiver how to administer the medication at home. The remaining doses will be administered by the caregiver. The amoxicillin routinely used by CSPS staff for the management of SAM is used for the study (amoxicillin syrup 250 mg/5ml, Reyoung Pharmaceutical, Shandong, China).

Except for antibiotics, all children will receive standard outpatient treatment for uncomplicated SAM per the guidelines of the government of Burkina Faso, which includes provision of ready-to-use therapeutic food (RUTF), anti-malarials if positive for malaria after rapid diagnostic test, anti-parasitics, missing vaccinations, and vitamin A.

### Adverse events

Parents or guardians of enrolled children will be instructed to report any adverse events experienced within the 7 days following the enrollment visit, by phone or in person. At all follow-up visits, study staff will inquire about the child’s experience of adverse events, including if the child had fever, diarrhea, vomiting, abdominal pain, skin rash, and/or constipation. The study team will ask whether the parent or guardian sought care for the child since the last visit and, if so, what the reason was for the health care visit and if the child was hospitalized.

Serious adverse events are defined as death, hospitalization, or any other life-threatening situation. Serious adverse events will be reported to the Medical Monitor within 24 h. The study team member conducting the follow-up visit will notify the site Principal Investigator, who will immediately notify the Medical Monitor and the UCSF study team. The Medical Monitor will determine whether the event could be reasonably considered to be related to the study drug and will report the results of this determination to the study investigators and the DSMC as needed. Information on all adverse events, serious and non-serious, will be recorded on data collection forms through the mobile application.

### Primary feasibility outcomes

- *Enrollment potential*. Enrollment will be tracked by enrollment site and date to monitor enrollment speed and progress over time. The average number of participants enrolled per site per day will be calculated and used to estimate the time required to enroll the target sample size for a fully powered randomized controlled trial. We will consider enrollment in a full-scale trial to be feasible if the sample size target is met during one malnutrition season (approximately a 5-month period from June through October).
- *Refusals*. The percentage of eligible participants refusing to participate will be calculated. We will consider success to be a refusal rate of < 10% of eligible participants. If refusals exceed this, we will investigate reasons for refusing study participation to better understand how to enhance recruitment.
- *Loss to follow-up*. The percentage of participants with incomplete follow-up at each time point will be calculated. We will consider success to be retention at the 8-week study visit of > 90% and will use data collected during the trial to refine retention procedures should loss to follow-up exceed this.
- *Completeness of data collection*. The percentage of variables with missing values will be calculated for each data collection form and time point. We will consider success for completeness of data collection to be < 1% of data collection forms with missing values.
- *Sample size assumptions*. Data on weight gain, nutritional recovery, and loss to follow-up will be monitored in both arms during the course of the trial. Sample size calculations for a fully powered trial will use these data from the amoxicillin arm in the pilot.

### Clinical outcomes

#### Primary clinical outcome

The primary clinical outcome for this trial is weight gain over the 8-week study period. Weight will be measured at baseline and all follow-up time points. Weight gain will be defined as grams per kilogram per day (g/kg/day).

#### Secondary clinical outcomes

- *Nutritional recovery by 8 weeks*. Nutritional recovery will be defined as a child having weight-for-height *Z*-score (WHZ) ≥ −2 on two consecutive visits and no acute complication or edema for the past 7 days OR MUAC ≥ 125mm on 2 consecutive visits and no acute complication or edema for the past 7 days. The criterion chosen to define recovery is the same one used to admit the child into the program.
- *Time to recovery*. Time from enrollment to nutritional recovery will be calculated in days by subtracting the date of enrollment from the date of nutritional recovery.
- *Nonresponse at 8 weeks*. Nonresponse will be documented if a child does not meet the criteria for nutritional recovery at 8 weeks.
- *Transfer to inpatient care*. The occurrence, date, and reason for transfer from outpatient to inpatient treatment will be recorded.
- *Mortality by 8 weeks*. Vital status will be assessed at all follow-up time points and mortality will be defined as death during the study period. Date of death will be recorded as applicable.
- *Clinical signs of infection*. At all study visits, clinical signs of infection will be recorded, including caregiver-reported experience of fever, diarrhea, vomiting, and/or respiratory infection/cough and clinical diagnoses made at by site personnel.
- *Adverse events*. Adverse events will be reported at all follow-up time points (defined in detail below).
- *Height-for-age Z-score (HAZ)*. Height or length will be measured at all study visits, and HAZ will be calculated using the World Health Organization Child Growth Standards. Height is assessed on children able to stand and length is assessed on children unable to stand.
- *Mid-upper arm circumference (MUAC)*. Mid-upper arm circumference will be measured using the nutritional program’s standard MUAC tape at all study visits.
- *Weight-for-age Z-score (WAZ)*. Weight will be measured at all study visits, and WAZ will be calculated using the World Health Organization Child Growth Standards.
- *Weight-for-height Z-score (WHZ)*. Weight and height will be assessed at all study visits and are used to calculate WHZ using the 2006 World Health Organization Child Growth Standards.
- *Malaria*. Rapid diagnostic tests for malaria will be conducted at baseline and week 8 to determine malaria infection status.
- *Intestinal microbiome*. Rectal swabs will be collected in a random subset of children at baseline and at week 8 and processed to evaluate the microbiome.
- *Anemia*. Hemoglobin will be assessed on a subset of children at baseline and at week 8 using a sample of blood from a finger or heel prick. Anemia will be defined as hemoglobin < 11 g/dl.

### Participant timeline, procedures, and follow-up

Figure 1 depicts the flow of participants through the study. After enrollment, a baseline assessment will be conducted which includes a questionnaire on socioeconomic status and feeding practices, anthropometry, and a rapid diagnostic test (RDT; SD Bioline Malaria Ag P.f./P.v., Abbott, USA) for malaria. Anthropometry includes an assessment of height using the ShorrBoard Infant/Child Measuring Board (Weight and Measure, LLC, USA), weight using the Seca 874dr scale (Seca, Germany), and MUAC using the Shorr Child MUAC tape (Weight and Measure, LLC, USA). Each anthropometric assessment will be taken three times and the median used for analyses.

The baseline visit will also include rectal swabs and hemoglobin assessments on subsets of enrolled children. A random sample of 100 children (50 children per arm) will be selected to receive a rectal swab at baseline. Rectal swabs will be collected by the study nurse and stored in RNA/DNA shield medium (Zymo Research, USA) at ambient temperature for no more than 5 days until they are stored in a −80°C freezer for long-term storage. An assessment of hemoglobin (HB301 analyzer; Hemocue, USA) is also conducted in children enrolled at 4 of the 6 enrollment sites using a sample of blood from a finger or heel prick.

Enrolled children will then be followed weekly at each routine follow-up visit until nutritional recovery and again at 8 weeks following admission. Each weekly visit will have a window of ±2 days. Anthropometry, vital status, adverse events, and clinical examination outcomes including diagnoses and treatments will be recorded at each weekly visit. The final study visit at 8 weeks will include these procedures as well as a rapid diagnostic test for malaria and hemoglobin as described above. At 8 weeks (−5 days, +21 days), the random subset selected at baseline will also receive another rectal swab. Study team members conducting procedures will undergo an initial 2-day training as well as refresher trainings as needed.

### Data collection and management

Data will be collected on enrolled children at baseline and follow-up visits during weeks 1–8 as outlined in Fig. 1. All data will be collected electronically on mobile devices using a custom-designed mobile application (CommCare by Dimagi, Cambridge, MA, USA). Data will be uploaded daily to a secure, password-protected, cloud-based server. All devices used for data collection will be password protected, as will the mobile application itself. Study data will only be accessible by study team members and investigators and de-identified datasets are prepared for analysis.

All team members collecting data will undergo an initial training to learn how to use the mobile devices and best practices for data collection. Weekly reports will be prepared by the UCSF data team to monitor study progress, data collection quality, follow-up, and adverse events. Concerns over data quality or completeness will be relayed to the local study team and resolved by email, with refresher trainings or additional supervision planned as needed.

### Sample size considerations

As the primary aim of this pilot trial is to establish feasibility, a sample size of 300 was chosen pragmatically to balance resource and logistical constraints with the objectives of determining the viability of a larger trial. Given this fixed sample size, power calculations were conducted for the primary clinical outcome, weight gain. Assumptions for these calculations were based on the pattern of weight gain and nutritional recovery reported in a trial of routine amoxicillin for uncomplicated SAM in Niger comparing children receiving amoxicillin to placebo over time [4]. Inclusion of 300 children (150 randomized to each arm) will provide 80% power to detect a 27% increase in weight gain (g/kg/day) in children receiving azithromycin compared to children receiving amoxicillin at an alpha of 0.05. Assumptions include an average weight gain of 4.9 g/kg/day in the amoxicillin arm, with a standard deviation of 3.9 g/kg/day, and loss to follow-up of 10%. A 27% increase corresponds to a mean difference in weight gain of 1.3 g/kg/day or an average weight gain of 6.2 g/kg/day in the azithromycin arm.

### Statistical analysis

Feasibility outcomes will be analyzed descriptively. These include calculation of enrollment speed, refusals, loss to follow-up, and completeness of data collection. These metrics will be summarized in aggregate and by arm. In addition, weight gain and nutritional recovery over 8 weeks by arm in this trial will be used to calculate the sample size required to conduct a full-scale trial in this setting. The target sample size calculation combined with the enrollment speed will be used to determine the feasibility of completing enrollment within a 2-year period.

The primary clinical outcome analysis will compare weight gain (g/kg/day) by arm over the 8-week period using a linear regression model, with similar secondary analyses comparing weight gain at interim time points. For secondary clinical outcomes, time to event outcomes will be assessed visually with Kaplan-Meier survival curves and compared by arm using a log-rank test and Cox proportional hazards regression. Binary outcomes will be analyzed using modified Poisson regression for rare outcomes (e.g., mortality) or log-binomial regression for common outcomes (e.g., nutritional recovery, malaria). If a log-binomial model fails to converge, modified Poisson regression will be used instead [15, 16]. Linear regression models will be used to compare anthropometric outcomes by arm at each time point with a term for the baseline measurement. Anthropometric outcomes will be analyzed as continuous variables and secondary analyses will explore categorization based on standard cutoffs for moderate and severe malnutrition. All analyses will be intention-to-treat and conducted in R (R Foundation for Statistical Computing, Vienna, Austria). A significance level of 0.05 for inference and 95% confidence intervals will be reported for all effect estimates.

### Dissemination plan

Results will be presented to local, national, and international audiences and prepared as manuscripts for publication.

## Discussion

With a high risk of infectious mortality, children with SAM stand to benefit from antibiotics, yet evidence on the impact of standard-of-care amoxicillin on mortality and nutritional outcomes is mixed. Azithromycin is a potential alternative to amoxicillin that could be administered as a single dose, obviating the need to rely on caregiver dosing over time. This pilot randomized controlled trial will establish preliminary evidence of the efficacy of azithromycin compared to amoxicillin to improve nutritional outcomes in children with uncomplicated SAM.

This pilot trial will determine the feasibility of a full-scale randomized controlled trial evaluating the role of antibiotics in the management of SAM. Despite the high burden of malnutrition and availability of experienced research staff at nutritional programs, no prior trial has been conducted on antibiotics in SAM management in Burkina Faso. This initial study will establish the potential speed of enrollment for a future trial. In addition, the pilot period allows for an assessment of the feasibility of the proposed study procedures, including the addition of a rectal swab and hemoglobin assessment to the routine clinical evaluation conducted in the CSPS nutritional programs. Finally, the sample size calculations for this pilot are based on data available from a prior trial conducted in Niger. The present study will allow us to refine these calculations using data from nutritional programs in Burkina Faso.

Given the small sample size, the pilot trial will have limited power to detect an impact on rare outcomes like mortality or small effects on important indicators like nutritional recovery. Similarly, the pilot will not be powered to detect effects in subgroups defined by baseline characteristics, like age or sex. As the assumptions for sample size calculations were based on a trial conducted in Niger several years ago, actual weight gain might be lower in this setting, potentially resulting in this study being underpowered to detect important differences across intervention arms. Lastly, given the limited resources available for the pilot, additional outcomes to be included in the future trial are not assessed, including more extensive assessment of antimicrobial resistance and nutritional biomarkers.

Overall, we anticipate the results of this pilot study to provide preliminary evidence and support future evaluations of the role of adjunctive antibiotics in the management of uncomplicated SAM.

## Trial status

The text of this manuscript refers to protocol version 3.3, 30 June 2020. Recruitment began on 3 June 2020 and enrollment was completed on 9 October 2020. Follow-up visits are currently in progress and are expected to continue through December 2020.

## Data Availability

Not applicable. Upon completion of the trial, de-identified data will be made available upon request.

## Abbreviations

CSPS: Centre de Santé et de Promotion Sociale
DSMC: Data and Safety Monitoring Committee
HAZ: Height-for-age *Z*-score
MORDOR: Macrolides Oraux pour Réduire les Décès avec un Oeil sur la Résistance
MUAC: Mid-upper arm circumference
RDT: Rapid diagnostic test
RUTF: Ready-to-use therapeutic food
SAM: Severe acute malnutrition
UCSF: University of California, San Francisco
WAZ: Weight-for-age *Z*-score
WHO: World Health Organization
WHZ: Weight-for-height *Z*-score
